# Echolocating bats emit a highly directional sonar sound beam in the field

**DOI:** 10.1098/rspb.2008.1505

**Published:** 2008-12-02

**Authors:** Annemarie Surlykke, Simon Boel Pedersen, Lasse Jakobsen

**Affiliations:** 1 Institute of Biology, University of Southern Denmark5230 Odense M, Denmark; 2 Skovvænget 232970 Hørsholm, Denmark

**Keywords:** echolocation, bat, directionality, *Myotis*, field, sonar

## Abstract

Bats use echolocation or biosonar to navigate and find prey at night. They emit short ultrasonic calls and listen for reflected echoes. The beam width of the calls is central to the function of the sonar, but directionality of echolocation calls has never been measured from bats flying in the wild. We used a microphone array to record sounds and determine horizontal directionality for echolocation calls of the trawling Daubenton's bat, *Myotis daubentonii*, flying over a pond in its natural habitat. *Myotis daubentonii* emitted highly directional calls in the field. Directionality increased with frequency. At 40 kHz half-amplitude angle was 25°, decreasing to 14° at 75 kHz. In the laboratory, *M. daubentonii* emitted less intense and less directional calls. At 55 kHz half-amplitude angle was 40° in the laboratory versus 20° in the field. The relationship between frequency and directionality can be explained by the simple piston model. The model also suggests that the increase in the emitted intensity in the field is caused by the increased directionality, focusing sound energy in the forward direction. The bat may increase directionality by opening the mouth wider to emit a louder, narrower beam in the wild.

## 1. Introduction

Bats use echolocation or biosonar for navigation and prey detection at night. They emit short, high-frequency calls and listen for echoes from background and prey. By adapting acoustic features such as frequency, duration and repetition rate of its outgoing echolocation sound, the bat has active control over the echo picture of the surroundings it perceives. A large number of studies have demonstrated how these features are shaped by the perceptual challenges in the natural environment (e.g. Griffin 1958; Neuweiler 1989; Schnitzler *et al*. 2003; Jones 2005). Two other acoustic features of the calls—intensity and directionality—are hardly ever reported from field studies, although they are just as significant for the echolocation, since they control the range and width of the bat's sonar. Measuring these data in the wild is a challenge, requiring determination of distance, direction and relative flight velocity of bats that move at high speeds in the dark. Therefore, intensity is most often left out (Kazial *et al*. 2008) and directionality has only been studied in the laboratory (Shimozawa *et al*. 1974; Schnitzler & Grinnell 1977; Hartley & Suthers 1987, 1989; Henze & O'Neill 1991; Hiryu *et al*. 2006). However, the field recordings of intensity (Griffin 1958; Jensen & Miller 1999; Holderied *et al*. 2005; Surlykke & Kalko 2008) indicate pronounced directionality by showing large intensity differences between on-axis and off-axis recordings. Thus, the intensity and directionality are linked, making it hard to estimate the intensity and nearly impossible to estimate the directionality in the field without using a system allowing for monitoring the acoustic axis such as, for example, the microphone array employed here. A highly directional echolocation call provides inherent directional information of echoes, attenuation of background and reduced energy expenditure. The importance of directionality of animal sound emissions is not limited to echolocation; it is essential for any kind of animal acoustic communication, since directionality of the emitted sound is critical for communication distance and direction (Dantzker *et al*. 1999).

Vespertilionid bats have simple faces and emit sounds through the mouth. Directionality of their sonar beam can be approximated by a simple piston model for sound radiation of a circular piston in an infinite baffle:

(1.1) $$R_{P}(\theta )=\left|\frac{2\cdot J_{1}(k\cdot a\cdot \text{sin}(\theta ))}{k\cdot a\cdot \text{sin}(\theta )}\right|,$$

where *R*_*P*_(*θ*) is the ratio between the sound pressure levels (SPLs) on-axis and off-axis at an angle *θ*; *J*_1_ is a first-order Bessel function (Morse 1948) with *k*=2*π*/*λ*; *λ* is the wavelength; and *a* is the radius of the piston (Strother & Mogus 1970). According to the model, the width of the sound beam is proportional to the wavelength relative to the diameter of the piston. Thus, directionality correlates positively with the size of the emitter and with the frequency of the sound. The piston model also predicts notches in the radiation pattern at specific angles. Such notches were measured by Mogensen & Møhl (1979) in the horizontal emission pattern of Daubenton's bat, *Myotis daubentonii*, hence corroborating the piston model for vespertilionid bats.

Mogensen & Møhl (1979) recorded calls from a bat fixed in a position by the skull and supported by a ball. The emitted calls were 3 ms frequency modulated (FM) sweeps from 50 to 25 kHz with main energy around 30 kHz, hence quite different from natural *M. daubentonii* search calls, which are steep broadband FM sweeps from 90 to 35 kHz lasting 3–5 ms (Kalko & Schnitzler 1989). Other measurements of directionality have also been done with bats sitting on a platform or otherwise restrained and in some cases calls were elicited by brain stimulation, such that in general the calls were different from natural search calls (Shimozawa *et al*. 1974; Grinnell & Schnitzler 1977; Hartley & Suthers 1989; Henze & O'Neill 1991). A more natural set-up was used by Ghose & Moss (2003), Moss *et al*. (2006) and Ghose *et al*. (2007), who trained big brown bats, *Eptesicus fuscus*, to capture prey on the wing in a large flight room, but even here the calls were less intense, shorter and of broader bandwidth than signals emitted in the field (Surlykke & Moss 2000). With spectral, temporal and intensity parameters all being different in the laboratory compared with the natural habitat, it is also likely that directionality is different between the laboratory and the field. Hence, it is essential for determining biologically relevant measures for directionality to study bats flying freely in their natural environment.

To facilitate determination of directionality in the field, we chose to study a trawling bat that hunts prey over water. Trawling bats fly at almost constant altitude above the water surface, i.e. mainly in only two dimensions, increasing the chance of recording bats at the height of the microphones. A trawling strategy has evolved independently in several bat families such as Vespertilionidae, Noctilionidae, Emballonuridae and Phyllostomidae (Weinberg & Kalko 2007). This may be due to the prey density just above water surfaces, or perhaps related to the acoustic properties of water (Siemers *et al*. 2001). As did Mogensen & Møhl (1979), we studied *M. daubentonii* (Vespertilionidae), but in the wild as they hunted insects over a local pond. We used a microphone array designed to determine the horizontal directionality of the echolocation signals. To our knowledge, this is the first study to establish directionality of echolocation sounds from bats flying freely in their natural habitat.

We predicted that Daubenton's bat emits highly directional calls. We further hypothesized that directionality will be more pronounced in the field than in the laboratory. We discuss the significance of directionality for the function of bat biosonar in biologically relevant situations.

## 2. Material and methods

### (a) Animals and recording sites

We recorded *M. daubentonii* hunting over a pond, Skovsøen, in Odense, Denmark, in September 2003 and October 2005. We attracted the bats by throwing mealworms on the water surface approximately 1.5 m in front of the microphones. In 2003 we recorded 130 files and, in 2005, 260 files with good signal-to-noise ratio on all microphones.

We also recorded calls from six *M. daubentonii* flying in the laboratory, a large flight room of 7×4.8×2.4 m at the University of Southern Denmark, Odense. The flight room was a net tent in a very large cellar room. The floor was covered with a carpet and had a water pool, 2.5×2.5 m, in the middle.

### (b) Sound recordings

**Figure 1:**
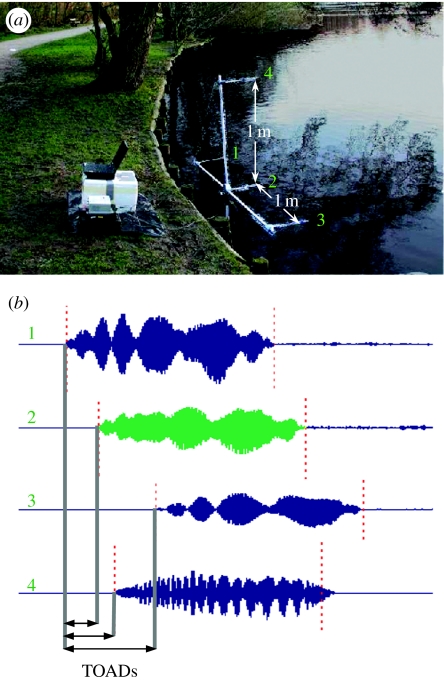
(*a*) The set-up with a linear horizontal array of three microphones and one microphone above the middle microphone. The microphones were separated by 1 m. The array was set with the horizontal microphones 30 cm over the water of a pond, where *M. daubentonii* hunted every night. The four microphones are marked with green numbers on the array and on the corresponding channels of the recording. We determined the (*b*) time-of-arrival differences (TOAD) of the sonar sounds between recording channels by cross-correlation.

In 2003, we used a linear array with three microphones, 1 m apart. In 2005, we added an extra microphone 1 m above the middle microphone in the array (figure 1). The three aligned microphones were 30 cm above the water at approximately 0.5 m horizontal distance from the brink. The microphones (1/4″ BF GRAS microphones without grids) were mounted on thin (5 mm) rods. Signals were amplified (GRAS 12AA, with custom-built 13 kHz high-pass (HP) filter) and recorded digitally (sampling rate 250 kHz per channel, eight order low-pass anti-aliasing filter with *f*_−3 dB_=110 kHz) using three or four channels on a Wavebook 512 (IOtech, Cleveland, OH, USA) A/D and stored on an IBM notebook computer, which was also used to check the recordings online. The Wavebook had 128 MB circulating buffer memory, allowing for manual post-triggering with delay set to 3 s. We only recorded bats approaching the array at an angle of approximately 30°.

In the laboratory, we recorded the signals on seven channels, using two simultaneously triggered Wavebooks to digitize the signals from an array with six 1/4″ microphones on a horizontal line 70 cm apart and one 1/4″ microphone 1 m above to control for flight height. The bats would circle the flight room, and recordings were manually triggered using the post-trigger system when they were approaching at the height of the horizontal array. Hence, each signal was recorded at six different horizontal angles simultaneously.

### (c) Sound analysis

The field recordings with good signal-to-noise ratio (S/N>+10 dB for signal energy relative to energy of the noise immediately before the signal) on all microphone channels were analysed signal by signal in the frequency and time domain using a custom-made signal analysis program, SigPro. Only search calls were included in the subsequent analyses. We used the microphone array recordings to reconstruct the flight paths, making use of the time-of-arrival differences (TOAD) between the microphones, which we found by cross-correlating the signals recorded on three or four channels (figure 1). From the TOAD we estimated the bat's position for each call. A linear array yields only two of the three coordinates required for absolute positioning of a source, i.e. a circle with its centre on the line through the microphones, and a radius equal to the distance to the source (Surlykke *et al*. 1993; Madsen & Wahlberg 2007). However, since the trawling bats always flew closely over the water surface, the three-dimensional positioning was unambiguous. This was confirmed by the recordings with the four-microphone array. The acoustic positioning method has been confirmed against a photographic method (Surlykke & Kalko 2008). The main reason for positioning error is inaccuracy in determining the delay between microphones, which is partly caused by the sampling and partly by Doppler shift due to the bat's relative velocity with respect to the microphones. By resampling the recordings at 1 MHz with appropriate filtering we improved the accuracy of the cross-correlation itself from 4 to 1 μs. Doppler shifts will introduce errors of up to approximately 10 μs for *M. daubentonii* with flight speed of approximately 4 m s^−1^, assuming that the search calls last for 3 ms and sweep from 95 to 35 kHz. Thus, in an extreme case where a bat flew along the array towards one microphone and away from another, the maximum Doppler-dependent error would be approximately 20 μs, which translates into positioning errors of up to 10 cm for bats 10 m from the array and 1–3 cm for bats close to the array.

**Figure 2:**
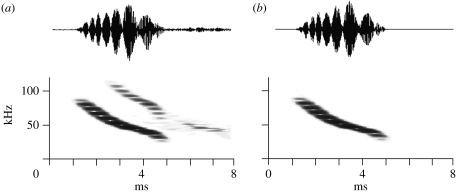
(*a*,*b*) Isolation of first harmonic. The echolocation calls were broadband FM sweeps with a prominent second harmonic. The first harmonic was isolated by harmonic filtering, which removed the interference in time signals and spectra that was due to the frequency overlap between first and second harmonic.

We compensated for transmission loss due to the distance to the bat, i.e. spherical spreading loss (−6 dB per doubling of distance) and frequency-dependent atmospheric attenuation at 16°C and 80 per cent relative humidity (ANSI 1978) corresponding to the average climatic conditions at the study site. We also compensated for the directionality of the 1/4″ microphones (Brüel & Kjær 1982). To determine the directionality of the first harmonic, we had to isolate it, but simple low-pass filtering was inadequate due to the frequency overlap between the first and second harmonic. A program employing a graphic method developed by Beedholm (2004) was used for harmonic filtering (figure 2). All SPLs are given as dB SPL relative to 20 μPa rms (root mean square).

### (d) Estimating directionality

We computed flight paths from a series of successive positions. We limited the analysis to calls emitted within 10 m from the array, because further away the positioning was not accurate enough (Madsen & Wahlberg 2007) and the directions from bat to microphones were too similar. Files with simultaneous recordings of more bats were not included. It was assumed that bats project their sonar beam in the direction they fly. Hence, the beam direction at a particular call was estimated as the average flight direction from the previous to the following call. We did not include calls where the flight path showed sharp turns before or after the call. From the bat's position and the sonar beam direction, we then calculated the numerical value of the attack angle to each microphone in the linear array (see §3). The calls were pooled in six angle groups from 0 to 60°. To calculate the average for each angle group, the spectra were normalized to 0 dB at 45 kHz and 0°. If a call was not recorded at 0°, the spectral value at 45 kHz at the lowest angle recorded was used as an anchor point.

We determined the directionality of the bats' emitted signals from the pooled averaged data within each angle group. In addition, we checked the procedure by determining directionality for single calls recorded simultaneously at different angles from multiple microphones to compare with the pooled data.

The signals recorded in the laboratory were compensated for transmission loss and microphone directionality, and subsequently pooled and averaged in the same way as the field data.

## 3. Results

### (a) Recordings

On a given night, between 5 and 15 *M. daubentonii* reliably hunted at the field site. They flew over the pond (approx. 200×80 m) and the river (Odense Å) next to the pond. After one night of throwing mealworms on the water, a number of bats (5–10) concentrated around the set-up, where a continued supply of mealworms kept them interested, thus increasing the number of good recordings. We estimated flight speeds from six flight paths, where the bat flew on a straight course over a number of search calls. The average flight speeds varied between 3.6 and 4.6 m s^−1^ with a mean of means of 4.0±0.4 m s^−1^. The duration of search calls was 4–5 ms. The calls were steep FM sweeps with a first harmonic covering a broad bandwidth from 90 to 35 kHz (figure 2), corroborating the results for *M. daubentonii* from other areas (Kalko & Schnitzler 1989; Britton & Jones 1999). After screening files with approximately 35 000 signals, we included a final number of 15 flight paths in the analysis and computed directionality from a total of 195 calls recorded on three or four microphones. There is no control for pseudo-replication in the data, but it is highly unlikely that all recordings were from the same individual bat, since the recordings were done over two years, and, while recording, we observed at least four to five bats hunting close to the microphones.

### (b) Spectra of emitted signals

**Figure 3:**
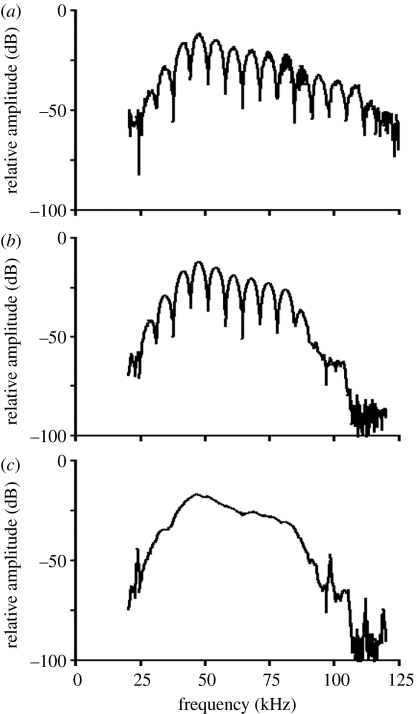
Removing interference from water reflections. The interference from the overlap on the microphone between the directly transmitted call and the reflection from the water surface created deep notches in the spectra. (*a*) The raw spectrum. (*b*) The spectrum of the first harmonic alone after harmonic filtering. We removed the reflections by a custom-made program, BatIron, and the resulting spectrum of the directly transmitted signal is shown in (*c*).

The interference between the directly transmitted signals and the reflection from the water surface created typical notches in the signal and spectrum (figures 1 and 3; Surlykke & Kalko 2008), making systematic determination of directionality from raw spectra impossible. We developed a program, BatIron, to mathematically remove the reflections from the water surface (figure 3). For numerical values, the spectrum of sound and reflection on the microphone, *R*_m_(*f*), is a function of *R*(*f*), the spectrum of the direct echolocation sound, and *α* and Δ*T*, where *α* is the reflection coefficient and Δ*T* the delay between direct and reflected signal:

(3.1) $$|R_{\text{m}}(f)|=|{\left[(1+\alpha^{2})+2\alpha \;\text{cos}(2\pi f\Delta T)\right]}^{1/2}||R(f)|.$$

The position and magnitude of the notches in the spectra were used to determine Δ*T* and *α*. The *α* values were between 0.85 and 0.95, not 1 (perfect reflection), even though a calm flat water surface reflects practically all sound. Directionality of the echolocation sound explains this, because the direct and reflected signals were ‘seen’ from different angles with respect to the bat. The reflected signal is recorded from an angle corresponding to a virtual microphone as far below the water surface as the real microphone is above the water surface, i.e. off-axis in the vertical plane.

The reflections were not only a problem, but also a source of information, because the recordings could be treated as if recorded by an array with twice as many microphones: three real and three virtual. The array with three (real) microphones thereby provided enough information to calculate flight heights. In search flight the bats flew 12±5 cm below the microphones, i.e. 18±5 cm above the water surface. Flight heights based on Δ*T*-values were confirmed by three-dimensional positioning with the four-microphone array.

### (c) Directionality

**Figure 4:**
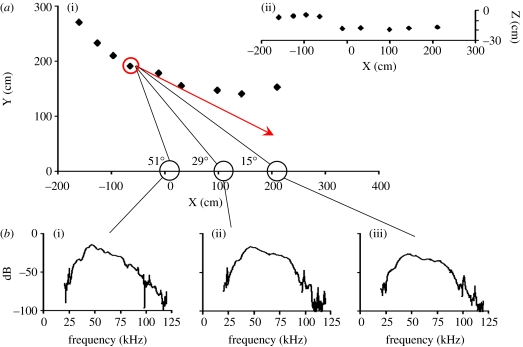
Flight path, beam direction and spectra. The bat's position at each call was determined from the TOAD and was used to estimate flight paths in (*a*(i)) the horizontal and (ii) vertical plane. The sonar beam axis was assumed to be parallel to the flight direction (red arrow). From the beam axis, we determined the attack angle (black lines) to each of the microphones in the horizontal array. From left to right, distance and attack angle for the call emitted at the position marked by the red circle were 202 cm, 51°; 252 cm, 29°; 327 cm, 15°. (*b*(i)–(iii)) The three spectra of this call recorded at the three microphones, illustrating how spectrum depends on distance and direction between bat and microphone.

**Figure 5:**
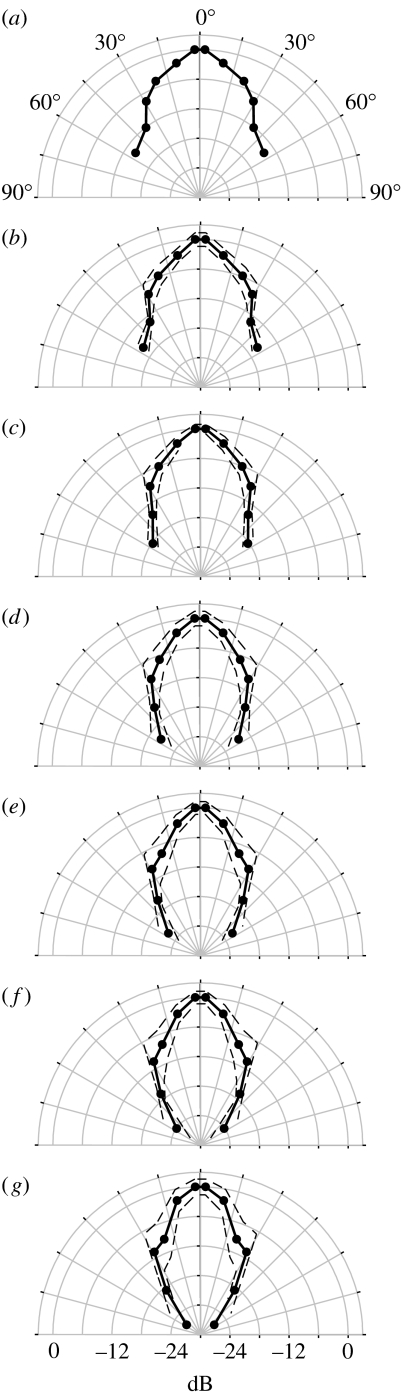
Echolocation beam shape depends on frequency. The directionality is shown as beam patterns at frequencies of (*a*) 45 kHz, (*b*) 50 kHz, (*c*) 55 kHz, (*d*) 60 kHz (*e*) 65 kHz, (*f*) 70 kHz and (*g*) 75 kHz. The mirror images of the values around 0° were added to create symmetrical plots. Standard deviations are shown as dashed curves. All plots illustrate how sound pressure decreases as off-axis angle increases. The plots also show how directionality increases with frequency.

We subtracted the reflections to get the spectra of the first harmonic as a function of the angle (figure 4), pooled the data in angle groups and determined the amplitude as a function of recording angle and frequency. The resulting beam patterns were displayed as polar plots showing the sound pressure at off-axis angles relative to the sound pressure directly ahead (figure 5). The plots illustrate how the sound pressure decreases as off-axis angle increases at all frequencies.

Determining the directionality by combining the data from a large number of recordings was necessary to get values at many angles, but might obscure individual variation. Also, assuming that the acoustic axis is equal to the flight direction might be a problem, since our own video recordings from the field and the laboratory, as well as many other results (e.g. Ghose & Moss 2003), clearly reveal how bats may quickly turn the head away from the flight course. However, most of the time the sonar beam is probably pointing in the flight direction, especially on straight parts of the flight path with no sudden turns before or after. Furthermore, we avoided most errors, where the bat looked away from the flight direction, by only including the data where the intensity was highest on the microphone, which was closest to the acoustic axis, as estimated from the flight path.

**Figure 6:**
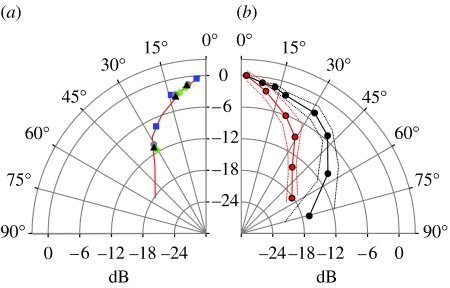
(*a*) Individual calls compared to average beam shape. Data points for three angles corresponding to the three microphones are shown from four individual recordings (squares, diamonds, circles and triangles) superimposed on the average beam pattern for 55 kHz (red curve), showing the fine correspondence between single data points and the averaged curve. (*b*) A comparison of directionality in the laboratory and the field at 55 kHz. In the laboratory, the sonar beam (black curve; standard deviation, dotted lines) was much broader than the beam emitted in the field (red curve; standard deviation, dotted lines). Half-amplitude angles at 55 kHz were 40° and 20° in the laboratory and the field, respectively.

To verify our averaged data, we analysed individual calls recorded simultaneously at the three horizontal microphones. The data points for these calls were superimposed on the average directionality plot at 55 kHz and these data entirely confirmed the averaged curve (figure 6*a*). This fine match between the averaged curve and individual calls, as well as the relatively low standard deviations (figure 5), confirmed our method. The plots show that the sound beam emitted by *M. daubentonii* is highly directional and that the directionality increases with frequency. Beam widths may be characterized by half-amplitude angle, i.e. the angle at a specific frequency, where the amplitude has decreased by 6 dB relative to 0°. At 45 kHz, the frequency with peak energy, the half-amplitude angle was 25°. At 55 kHz, the half-amplitude angle was 20° decreasing to 14° at 75 kHz (table 1).

We estimated the emitted intensity from echolocation calls that were recorded on-axis, i.e. at less than 5° off-axis. The intensity was calculated as source level (SPL referenced to 10 cm from the bat's mouth) by adding transmission loss to the recorded sound level (see Surlykke & Kalko 2008). In the field, the average source level was 119±4 dB SPL (rms).

*Myotis daubentonii* flying in the laboratory emitted signals that were of similar bandwidth but shorter duration, approximately 2.5 ms, than in the field. For each of the six bats, we determined directionality at 55 kHz from two echolocation signals from two different flights towards the array. The increased number of microphones in the laboratory allowed for more angle groups and measurements up to 75° off-axis. The calls were significantly less directional in the laboratory than in the field (*p*<0.01, *t*-test at 30°, 40°, 55°; figure 6*b*). At 55 kHz, the half-amplitude angle was 40°, i.e. twice the half-amplitude angle of 20° at 55 kHz in the field.

The emitted intensity was lower in the laboratory than in the wild. The average source level for calls recorded on-axis was 111±4 dB SPL (rms).

## 4. Discussion

We have here shown that the echolocation calls emitted in the field by *M. daubentonii*, and possibly many other species, are far more directional than previously assumed. We have also demonstrated that when bats fly in the laboratory, they decrease both the intensity and the directionality of their signals compared to when flying in the field.

The narrow sonar beam we report here for *M. daubentonii* flying in the field disagrees with the earlier laboratory results, which all indicated that bats, in spite of species differences, emit beams that are fairly broad. Half-amplitude angles of between approximately 30° and 50° have been reported for *Pteronotus parnellii* (Henze & O'Neill 1991), *E. fuscus* (Ghose & Moss 2003) and *Hipposideros terasensis* (Hiryu *et al*. 2006). Mogensen & Møhl (1979), also working with *M. daubentonii*, but under very unnatural conditions, reported a half-amplitude angle of approximately 38° at 55 kHz for a sitting restrained bat. Interestingly, even though our bats were not fixed in position, but flying freely in the laboratory, we found a similar half-amplitude angle of 40° at 55 kHz. In the field, *M. daubentonii* emitted a much narrower beam, with half-amplitude angle of only 20° at 55 kHz. The search signals were also more intense: approximately +8 dB louder in the field than in the laboratory.

From the half-amplitude angles at 55 kHz in the field and the laboratory, we calculated the equivalent piston radii from equation (1.1). Subsequently, we used these piston radii to calculate the corresponding directivity indices, DI=20·log(*k*·*a*) (Strother & Mogus 1970). At 55 kHz, the sound beam measured in the field has an equivalent piston radius of 6.4 mm and a DI of 16.2 dB, whereas in the laboratory the sound beam has an equivalent piston radius of 3.4 mm and a DI of 10.7 dB. Thus, according to the piston model, the increase in directionality, and hence the concentration of signal energy in the forward direction, will increase the on-axis source level by 5.5 dB, suggesting a link between the increase in directionality and the increase in source level. Whereas the model cannot explain the complete structure of echolocation beams from bats, e.g. that *E. fuscus*'s beam is bilobed in the vertical plane (Ghose *et al*. 2007), it does indicate that a part of the increase in source level in the wild is due to an increase in directionality, corresponding to an increase in equivalent piston diameter, for *M. daubentonii* from 3.4 to 6.4 mm. Directionality of a piston increases with diameter relative to wavelength. Thus, the bats may simply achieve increased directionality of their sounds by opening the mouth wider.

Source levels measured in the wild for Daubenton's bat surpass the laboratory values by 8 dB, i.e. by more than is explained by the increased directionality. Other studies have invariably shown that bats in the wild emit intensities far exceeding those estimated in the laboratory (Surlykke *et al*. 1993; Jensen & Miller 1999; Holderied *et al*. 2005; Surlykke & Kalko 2008). Hence, it is likely that bats also increase the power output in the field; but our results here indicate that the higher intensity is caused, at least to some extent, by increased directionality of sonar beams in the natural environment. This suggests that bats' attended sonar angle may not be as broad as the laboratory results imply (Ghose & Moss 2003). If the beam is broad, almost equal sound energy impinges on objects within a large angle, which would return concurrent audible echoes from many directions. Our data suggest that bats in the field ensonify and listen to the echo objects from a more narrow cone ahead of them. The width of the sonar cone significantly influences the three-dimensional representation of their immediate environment that bats get from echoes generated by objects in their surroundings.

Recordings from echolocating sperm whales indicate a correlation between intensity and directionality, with more intense signals being more directional (Møhl *et al*. 2000). However, adjustment of directionality in odontocetes seems to be through adjusting the emitted frequency, which is coupled to the emitted intensity. Generating high source level clicks with higher frequencies will produce a more narrow transmission beam (Madsen *et al*. 2004). In comparison, bats' active control over mouth opening gives them a higher degree of freedom to adjust directionality and source level of the transmitted beam independent of the emitted frequency. This ability to adapt range and width of the sonar contributes to the acoustic flexibility of bats, which is probably an important reason for the extraordinary diversity of echolocating bats, with more than 950 extant species exploiting a wide variety of food items and habitats. Here, we have focused on search calls, but the flexibility verified by the difference in directionality between the laboratory and the field suggests that bats may change the beam width through the phases of a pursuit to adapt the directionality as the task changes from detection to localization of the prey.

Bat echolocation frequency is assumed to be a compromise between resolution and range, because only at high frequencies (short wavelengths) will small insects be efficient echo reflectors (Møhl 1988), while at the same time atmospheric attenuation is also more severe as the frequency increases. However, the range reduction by increased atmospheric attenuation is offset by the increased directionality at higher frequencies, which focus more sound energy in the forward direction. This implies that the cost of increasing the frequency may not be the range, but rather the width, of the sonar, or the bat's ‘peripheral vision’.

Bats have been shown to respond to sonar calls of other bats up to 50 m away (Barclay 1982), supporting the idea that echolocation is also used for communication. Maximum communication distance will depend significantly on directionality. Indeed, directionality is important for all animals communicating by sound. While some findings may be specifically related to the special situation for echolocators (bats and odontocetes), the relationship between directionality, frequency and projected intensity will apply to the constraints for acoustic interaction in all animals. For ‘private’ communication, highly directional signals reduce the chance of others listening in on the ‘conversation’, while for territorial advertisement, omnidirectional vocalizations may maximize the number of neighbours that receive the message. It requires complicated equipment and intensive data analysis to assess directionality of sounds recorded in the field. Thus, only a few studies on any animal address the directionality of acoustic radiation (e.g. Dantzker *et al*. 1999; Møhl *et al*. 2000; Rasmussen *et al*. 2004; Patricelli *et al*. 2007, 2008). Bats are ideal animals for studying acoustic perception, because they rely so heavily on sound for orientation and communication and because their intense echolocation calls allow us to ‘eavesdrop’ on their emitted signals to uncover how they react to biologically relevant challenges.

In conclusion, we have shown here that it is possible to determine directionality from freely flying bats hunting in the field. The results revealed much higher directionality of bat sonar signals than previously assumed, which is of major importance for the range and width of the sonar, for the acoustic ‘scene’ the bats perceive through echolocation, and for the interaction and disturbance of close-by sympatric bats. We have also shown a substantial difference between the directionality of signals recorded in the laboratory and the field, emphasizing the value of studying animals in their natural habitat.

## Figures and Tables

**Table 1:** Half-amplitude angles. (The table shows the off-axis angle at which the signal amplitude has decreased by 6 dB compared with on-axis, 0°, for frequencies between 45 and 75 kHz.)

frequency (kHz)	45	50	55	60	65	70	75
half-amplitude angle (°)	25	22	20	18	17	16	14

## References

[bib1] ANSI (American National Standards Institute) (1978). Method for the calculation of the absorption of sound by the atmosphere.

[bib2] Barclay R.M.R. (1982). Interindividual use of echolocation calls, eavesdropping by bats. Behav. Ecol. Sociobiol.

[bib3] Beedholm, K. 2004 Aspects of signals and signal processing in echolocation by FM-bats for target range. See http://w210.ub.uni-tuebingen.de/dbt/volltexte/2004/1150/pdf/Beedholm.pdf

[bib4] Britton A.R.C., Jones G. (1999). Echolocation behaviour and prey-capture success in foraging bats: laboratory and field experiments on *Myotis daubentonii*. J. Exp. Biol.

[bib5] Brüel & Kjær (1982). Condenser microphones and microphone preamplifiers for acoustic measurements. Data handbook.

[bib6] Dantzker M.S., Deane G.B., Bradbury J.W. (1999). Directional acoustic radiation in the strut display of male sage grouse *Centrocercus urophasianus*. J. Exp. Biol.

[bib7] Ghose K., Moss C.F. (2003). The sonar beam pattern of a flying bat as it tracks tethered insects. J. Acoust. Soc. Am.

[bib8] Ghose K., Moss C.F., Horiuchi T.K. (2007). Flying big brown bats emit a beam with two lobes in the vertical plane. J. Acoust. Soc. Am.

[bib9] Griffin D.R. (1958). Listening in the dark.

[bib10] Grinnell A.D., Schnitzler H.-U. (1977). Directional sensitivity of echolocation in the Horseshoe bat, *Rhinolophus ferrumequinum*. II Behavioral directionality of hearing. J. Comp. Physiol. A.

[bib11] Hartley D.J., Suthers R.A. (1987). The sound emission pattern and the acoustical role of the noseleaf in the echolocating bat, *Carollia perspicillata*. J. Acoust. Soc. Am.

[bib12] Hartley D.J., Suthers R.A. (1989). The sound emission pattern of the echolocating bat, *Eptesicus fuscus*. J. Acoust. Soc. Am.

[bib13] Henze D., O'Neill W.E. (1991). The emission pattern of vocalizations and directionality of the sonar system in the echolocating bat, *Pteronotus parnelli*. J. Acoust. Soc. Am.

[bib14] Hiryu S., Katsura K., Lin L.-K., Riquimaroux H., Watanabe Y. (2006). Radiation pattern of echolocation pulse in Taiwanese leaf-nosed bat, *Hipposideros terasensis*. Acoust. Sci. Technol.

[bib15] Holderied M.W., Korine C., Fenton M.B., Parsons S., Robson S., Jones G. (2005). Echolocation call intensity in the aerial hawking bat *Eptesicus bottae* (Vespertilionidae) studied using stereo videogrammetry. J. Exp. Biol.

[bib16] Jensen M.E., Miller L.A. (1999). Echolocation signals of the bat *Eptesicus serotinus* recorded using a vertical microphone array: effect of flight altitude on searching signals. Behav. Ecol. Sociobiol.

[bib17] Jones G. (2005). Echolocation. Curr. Biol.

[bib18] Kalko E.K.V., Schnitzler H.-U. (1989). The echolocation and hunting behavior of Daubenton's bat, *Myotis daubentoni*. Behav. Ecol. Sociobiol.

[bib19] Kazial K.A., Pachecho S., Zielinski K.N. (2008). Information content of sonar calls of little brown bats (*Myotis lucifugus*): potential for communication. J. Mamm.

[bib21] Madsen P.T., Wahlberg M. (2007). Recording and quantification of ultrasonic echolocation clicks from free-ranging toothed whales. Deep-Sea Res. I.

[bib20] Madsen P.T., Kerr I., Payne R. (2004). Echolocation clicks of two free-ranging, oceanic delphinids with different food preferences: false killer whales *Pseudorca crassidens* and Risso's dolphins *Grampus griseus*. J. Exp. Biol.

[bib22] Mogensen F., Møhl B. (1979). Sound radiation patterns in the frequency domain of cries from a Vespertilionid bat. J. Comp. Physiol. A.

[bib23] Møhl B., Nachtigall P.E., Moore P.W.B. (1988). Target detection by echolocating bats. Animal sonar.

[bib24] Møhl B., Wahlberg M., Madsen P.T., Miller L.A., Surlykke A. (2000). Sperm whale clicks: directionality and source level revisited. J. Acoust. Soc. Am.

[bib25] Morse P.M. (1948). Vibration and sound.

[bib26] Moss C.F., Bohn K., Gilkenson H., Surlykke A. (2006). Active listening for spatial orientation in a complex auditory scene. PLoS Biol.

[bib27] Neuweiler G. (1989). Foraging ecology and audition in echolocating bats. Trends Ecol. Evol.

[bib28] Patricelli G.L., Dantzker M.S., Bradbury J.W. (2007). Differences in acoustic directionality among vocalizations of the male red-winged blackbird (*Agelaius pheoniceus*) are related to function in communication. Behav. Ecol. Sociobiol.

[bib28a] Patricelli G.L., Dantzker M.S., Bradbury J.W. (2008). Acoustic directionality of red-winged blackbird (*Agelaius phoniceus*) song relates to amplitude and singing behaviours. Anim. Behav.

[bib29] Rasmussen M.H., Wahlberg M., Miller L.A. (2004). Estimated transmission beam pattern of clicks recorded from free-ranging white-beaked dolphins (*Lagenorhyncus albirostris*). J. Acoust. Soc. Am.

[bib30] Schnitzler H.-U., Grinnell A.D. (1977). Directional sensitivity of echolocation in the horseshoe bat, *Rhinolophus ferrumequinum*. I Directionality of sound emission. J. Comp. Physiol. A.

[bib31] Schnitzler H.-U., Moss C.F., Denzinger A. (2003). From spatial orientation to food acquisition in echolocating bats. Trends Ecol. Evol.

[bib32] Shimozawa T., Suga N., Hendler P., Schuetze S. (1974). Directional sensitivity of echolocation system in bats producing frequency-modulated signals. J. Exp. Biol.

[bib33] Siemers B.M., Stilz P., Schnitzler H.-U. (2001). The acoustic advantage of hunting at low heights above water: behavioral experiments on the European ‘trawling’ bats *Myotis capaccinii*, *M. dasycneme* and *M. daubentonii*. J. Exp. Biol.

[bib34] Strother G.K., Mogus M. (1970). Acoustical beam patterns for bats: some theoretical considerations. J. Acoust. Soc. Am.

[bib35] Surlykke A., Kalko E.K.V. (2008). Echolocating bats cry out loud to detect their prey. PLoS ONE.

[bib37] Surlykke A., Moss C.F. (2000). Echolocation behavior of big brown bats, *Eptesicus fuscus*, in the field and the laboratory. J. Acoust. Soc. Am.

[bib36] Surlykke A., Miller L.A., Møhl B., Andersen B.B., Christensen-Dalsgaard J., Jørgensen M.B. (1993). Echolocation in two very small bats from Thailand: *Craseonycteris thonglongyai* and *Myotis siligorensis*. Behav. Ecol. Sociobiol.

[bib38] Weinberg M., Kalko E.K.V. (2007). Ecological niche and phylogeny: the highly complex echolocation behavior of the trawling long-legged bat *Macrophyllum macrophyllum*. Behav. Ecol. Sociobiol.

